# The Genus *Allium* as Poultry Feed Additive: A Review

**DOI:** 10.3390/ani9121032

**Published:** 2019-11-26

**Authors:** Damini Kothari, Woo-Do Lee, Kai-Min Niu, Soo-Ki Kim

**Affiliations:** 1 Department of Animal Science and Technology, Konkuk University, Seoul 05029, Korea; damini.kth@gmail.com (D.K.); caw147@naver.com (W.-D.L.); 2 Institute of Biological Resource, Jiangxi Academy of Sciences, Nanchang 330029, China; niulele88@126.com

**Keywords:** *Allium*, feed additive, beneficial effects, organosulfur compounds, polyphenols, poultry

## Abstract

**Simple Summary:**

The routine and unregulated use of in-feed antibiotics as growth promoters in poultry have been linked to the development of antimicrobial resistance, a serious global threat to the human, animal, and environment health. Growing public health concerns about food and environmental safety intensified the search for effective antibiotic alternatives in poultry production. The aim of this review is to present the current state of knowledge on the use of alliums as effective poultry feed additives in relation to their effects on growth performance, disease infections, gut and immune modulation, and product quality.

**Abstract:**

The genus *Allium*, belonging to the family Amaryllidaceae has been known since ancient times for their therapeutic potentials. As the number of multi-drug resistant infections has increased due to in-feed antibiotic usage in poultry, the relevance of alliums as feed additives has been critically assessed. Garlic and the other *Allium* species, such as onions, leek, shallot, scallion, and chives, have been characterized to contain a plethora of bioactive compounds such as organosulfur compounds, polyphenols, saponins, fructans, and fructo-oligosaccharides. Consequently, alliums have been validated to confer antioxidant, antibacterial, antiviral, immunostimulatory, gut homeostasis, and lipid- as well as cholesterol-lowering properties in poultry. This review intends to summarize recent progress on the use of edible alliums as poultry feed additives, their beneficial effects, and the underlying mechanisms of their involvement in poultry nutrition. Perspectives for future research and limitations are also briefly discussed.

## 1. Introduction

The prolonged and unregulated use of antibiotics driven by a growing demand for animal products lead to the emergence of antibiotic resistance, a global threat to the animal and human health [1,2,3]. Poultry is the world’s primary source of animal protein and it represents one of the highest consumers of antibiotics as growth promoters [3]. The European ban on sub-therapeutic use of antibiotics (1831/2003/EC, 2006) and the growing awareness among the consumers of the fatalistic effects of antibiotic resistance as well as residues in animal products intensified the hunt for effective in-feed antibiotic surrogates without affecting animal productivity or product quality [4,5,6]. However, the major challenges associated with antibiotic-free poultry production are poor growth performance, lower productivity, and increased morbidity as well as mortality in birds [7,8]. Many reviews shedding light on efficient and cost-effective antibiotic alternatives in poultry have been published in recent times [2,6,9,10,11]. Recently, plant-derived feed additives have gained considerable interest as sustainable substitutes in poultry diets [12,13]. An effective plant-derived additive in poultry (broilers, layers, and quails) is expected to stimulate feed intake, improve digestive enzyme secretions, activate immune system, modulate gut microbiota, as well as have antibacterial, coccidiostatical, antiviral, antioxidant and/or anti-inflammatory activities [12,13,14]. In this context, *Allium* holds immense promise due to a variety of bioactive compounds including organosulfur compounds (OSCs), polyphenols, saponins, fructans, fructo-oligosaccharides (FOS), among many others. The genus *Allium* of the Amaryllidaceae family consists of ca. 850 species and represents one of the most studied plants of medicinal importance [15]. Extensive literature is available on the therapeutic properties of *Allium* spp. in humans, however, there is poor evidence in the poultry counterpart. 

In the last three decades, alliums, in particular onion (*A. cepa*) and garlic (*A. sativum*), as well as garlic chives (*A. hookeri*) more recently have been reported to be incorporated into poultry diets to investigate their effects. However, the published literature on the effects of allium feeding in poultry have generated great inconsistency, making it impossible to draw a generalized conclusion on the efficacy of such feed additives. The discrepancies may be due to the heterogeneity of the composition of allium preparations, subject recruitment (broiler, layers, quails, etc.), dosage, duration of study, and so forth. This review combs the existing literature and gleans information to present an updated relevance of *Allium* spp. as effective poultry feed additives. We discuss the vast array of allium compounds in relation to their bio-functionalities. Emphasis was given to the dietary effect of *Allium* spp. on growth performance, infectious diseases, immunomodulatory properties, gut microbiota as well as gut morphology, and product quality in poultry. Moreover, this review discusses the lacunae to be surmounted for optimal application of alliums in poultry.

## 2. Overview of Major Bioactive Compounds in *Allium*

### 2.1. Organosulfur Compounds

**Figure 1:**
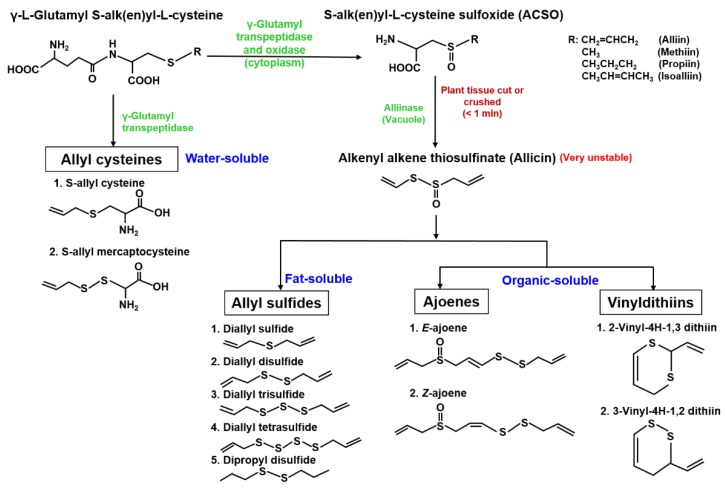
Major organosulfur compounds (OSCs) of *Allium* spp.

The genus *Allium* is a rich source of organosulfur compounds (OSCs), which are one the main bioactive compounds of the plants [16,17]. The major OSCs in *Allium* spp. include allyl cysteines, S-alk(en)yl-L-cysteine sulfoxides (ACSOs), thiosulfinates, and sulfides in varying amounts [18]. The characteristic aroma in different *Allium* spp. are mainly associated with the different levels of ACSO precursor, namely alliin (S-allyl-L-cysteine sulfoxide; garlic and elephant garlic), methiin (S-methyl-L-cysteine sulfoxide; garlic, onions, leeks, and shallots), propiin (S-propyl-L-cysteine sulfoxide; shallots), and isoalliin (S-1-propenyl-L-cysteine sulfoxide; onions and shallots) [19,20]. The synthesis of the OSCs is depicted in Figure 1 and starts with the transformation of γ-glutamyl peptides into ACSOs by the action of γ-glutamyl transpeptidase and oxidase in the cytoplasm of plant cells. When the bulbs are cut or crushed alliin is transformed into the allicin (alkenyl alkene thiosulfinate) by the action of a vacuolar lyase, alliinase. Allicin immediately decomposes into diallyl sulfide (DAS), diallyl disulfide (DADS), diallyl trisulfide (DATS), diallyl tetrasulfide (DATTS), dipropyl disulfide (DPDS), ajoenes, and vinyldithiins depending on their manufacturing process [21,22]. The direct catabolism of γ-glutamyl cysteine leads to the formation of water-soluble S-allylcysteine (SAC) and S-allylmercaptocysteine (SAMC) [23]. The OSCs and their transformation products are well-studied antimicrobial agents [24]. Several antimicrobial compounds have been extracted and identified from many spp. of *Allium* including garlic (*A. sativum* L.), onion (*A. cepa* L.), shallot (*A. ascalonicum* L.), elephant garlic (*A. ampeloprasum* L. var. *ampeloprasum* auct.), rosy garlic (*A. roseum*) [24], garlic chives (*A. hookeri*) [25], and wild garlic (*A. ursinum*) [26]. Although the antimicrobial mechanism of these compounds has not been well defined, it seems that it is associated with the inhibition of important thiol-dependent enzymatic systems (alcohol dehydrogenase, thioredoxin reductase, trypsin, other proteases, RNA and DNA polymerases) and antioxidant activity, which have a multiple inhibitory effect on the microbial cell [27,28,29]. The potent antimicrobial activities of OSCs is also related to the number of disulfide bounds, i.e., DATTS > DATS > DADS > DAS [30].

### 2.2. Polyphenolic Compounds

Another important class of bioactive compounds in alliums includes polyphenols [31,32]. The health-promoting activity of dietary polyphenols seems to be related to their antioxidant and anti-inflammatory activities [33]. Allium vegetables contain high levels of polyphenolic compounds, particularly phenolic acids, flavonoids, and their derivatives. *Allium* spp. are amongst the richest sources of dietary flavonoids [34]. Leighton et al. [35] found that flavonoid levels in the edible portion of allium vegetables (leeks, shallots, green onions, garlic, and onions) range from > 0.03 to 1 g/kg of vegetables. Flavonoids identified in onions were quercetin di-glucosides, quercetin 4‘-glucoside, quercetin aglycone, and in some cases, isorhamnetin monoglucosides or kaempferol monoglucosides [36]. Quercetin glucosides of onion are more bioavailable than other quercetin-rich foods such as tea and apples [37]. The main phenolic acids found in alliums include *p*-Coumaric acid, ferulic acid, sinapic acid, gallic acid, and protocatechuic acid [38]. However, very few studies used allium flavonoids as feed additives to promote growth, immune, and antioxidant response for animals [39,40].

### 2.3. Saponins

Saponins are surface-active glycosides with triterpenoid or steroidal aglycone. Allium plants contain steroidal saponins, which are mainly divided into three groups based on their structure: spirostanols, furostanols, and open-chain (cholestane-type) saponins [41]. Saponin accumulation in the root organs is reported to be higher than in the aerial parts (stem and leaves) of alliums [42]. Until now, as many as 290 steroidal saponins (130 spirostanols, 140 furostanol, and 18 cholestane-type) have been identified in more than 40 different *Allium* species [41]. Allium saponins are not pungent and have many biological properties including antispasmodic, antifungal, haemolytic, anti-inflammatory, cholesterol-lowering, and cytotoxic activities. Moreover, saponins have the advantage of being more stable to food processing and cooking than the relatively unstable OSCs [43].

### 2.4. Fructans and Fructo-Oligosaccharides

Water-soluble fructans and fructo-oligosaccharides (FOS) together with glucose, fructose, and sucrose constitute the main non-structural carbohydrates in *Allium* species [44]. Fructans from various spp. of *Allium* including *A. cepa* (onion), *A. cepa* L. var. *ascalonicum* (shallot), *A. ampeloprasum* L. var. *porrum* (leek, 3 cvs.), *A. schoenoprasum* L. (chives), *A. sativum* L. (garlic), *A. fistulosum* L. (Japanese bunching onion/Welsh onion), *A. tuberosum* Rottl. ex. spr. (Chinese chives) have been characterized [44]. Several in vitro and in vivo studies witnessed the immunomodulatory [45,46,47], prebiotic [48], antiviral [49], and gastroprotective [50] effects of allium poly- and oligosaccharides. Lee et al. [46] reported the influenza A virus inhibitory activity of the fructan from *A. fistulosum* in an animal model and it was suggested to be mediated by host immune functions since the polysaccharide did not show any direct inhibitory effect on the virus replication in vitro. The immunomodulatory effect was attributed to promotion of phagocytosis, release of NO, and expressions of several immune-related cytokines [interleukin (IL), tumor necrosis factor alpha (TNF-α), and interferon gamma (IFN-γ)] [47,48,51].

## 3. *Allium* spp. as Poultry Feed Additives

Literature search was conducted to accumulate the latest findings in implication of alliums as poultry feed additives and their role in growth performance, lipid metabolism, poultry infectious diseases, immunomodulation, gut modulation, and product quality (Table 1). The sections below outline the key components and mechanisms responsible for these functions.

### 3.1. Effects on Growth Performance

Several studies have documented the benefits of *Allium* spp. (in particular onion and garlic) on growth performance in poultry by improving weight gain, feed intake, and/or feed efficiency [52,53,54]. Farhani et al. [52] found that onion extract (1%) in drinking water improved growth performance and blood biochemical characteristics. They attributed the effect to the onion FOS, which might help in maintaining beneficial gut microorganisms and improve nutrient absorption. Goodarzi et al. [54] speculated that the OSCs of onion have increased nutrient absorption and thereby improved growth performance in broilers. In addition, onion in diet can reduce blood glucose stimulating the nervous system for higher feed intake, which can lead to increased weight gain [52,54]. 

The precise mechanisms behind the improved growth performance in poultry fed alliums remain unclear. However, some researchers have linked this improvement to the increased feed intake of allium supplemented diets [55]. Generally, garlic is used as seasonings to improve the flavor and hence it might improve the palatability of feed, thus increasing voluntary feed intake. Brzóska et al. [55] reported that garlic extract (2.25 mL/kg of feed) stimulated the appetite of chickens, which resulted in significantly greater feed intake and thereby higher body weight gains. Sheoran et al. [56] and Kirubakaran et al. [57] hypothesized that the improvement in weight gain of the birds using garlic in their rations may probably be due to allicin. Kirubakaran et al. [57] postulated that garlic in broiler diet may increase salivary flow rate and gastric juice secretion, resulting in improved digestibility and higher body weight. Negative effects on growth performance in broilers were also observed with the supplementation of 1 g of garlic powder/kg feed and 15 g of garlic bulb/kg feed [58,59]. The inclusion of alliums may reduce diet palatability due to their pungency and as a result the feed intake and body weight of animals decrease [58,60,61]. While Aji et al. [62] reported ineffectiveness of low doses (0.025 and 0.05 g of onion and garlic/kg feed) to produce any observable effects and suggested that the dosage of alliums as an important factor. However, several studies reported no significant effects on growth performance parameters such as feed intake, body weight gain, or feed efficiency in broilers by the dietary supplementation of alliums [14,60,62,63,64]. In the case of layers, most of the studies found no significant changes in performance when layer diets were supplemented with alliums [65,66,67,68]. Some researchers suggested that well-nourished healthy poultry reared under clean and ideal conditions, often do not respond to growth-promoting supplements, while the stressed or challenged birds may give better results with the same supplements [49,66,67,69,70,71,72]. Intriguingly, Ao et al. [66] indicated garlic supplementation could increase growth performance in broilers by reducing the concentration of cortisol, the stress hormone. The variability in the efficacy of alliums on animal performance could also be attributed to the variation in the product fed, dosage, duration, and subjects used among the studies.

### 3.2. Hypolipidemic and Hypocholesterolemic Effects

Alliums (especially garlic) have a traditional place in folk medicine as hypolipidemic and hypocholesterolemic agents in many cultures. Elevated blood cholesterol and triacylglycerides in animal proteins are associated with the increased risk of cardiovascular disease in humans. Several researchers have opined that garlic exhibited hypocholesterolemic effects in poultry including broilers, layers, and quails through the inhibition of key enzymes such as malic enzyme, fatty acid synthase, glucose-6-phosphate dehydrogenase, and 3-hyydroxy-methyl-glutaryl-CoA (HMG-CoA) reductase involved in cholesterol and lipid synthesis [81,83,85,86,87]. Allicin is thought to be the potentially active component [81,87]. However, Lanzotti et al. [43] reported that allicin is very unstable and not present in intact garlic or in any garlic products. Moreover, the acidity of the stomach prevents the formation of allicin [88]. Some researchers attributed the cholesterol-lowering effect of garlic to the steroidal saponins possibly by inhibiting cholesterol absorption in the intestine or a direct effect on cholesterol metabolism [41]. The exact mechanism by which garlic reduces plasma cholesterol concentration is remaining elusive.

### 3.3. Effects on Infectious Diseases

Recently, allium derived feed additives have been given useful results against several infectious diseases in broilers such as the infection of *Escherichia coli*, *Salmonella*, *Clostridium perfringens*, and *Eimeria* [27,71,72,89,90,91]. Elmowalid et al. [89] reported that garlic dietary supplementation for three weeks provided in vivo protection against multi-drug resistant *E. coli* O78 challenge in broilers by reducing the mortality rates to >10% from 60% (control, non-supplemented birds). The authors suggested that the bioactive phenolic and non-phenolic compounds in garlic are responsible for this effect. Lee et al. [71] reported less loss of body weight gain, decreased lesion score, and oocyst shedding by *A. hookeri* dietary supplementation in necrotic enteritis (NE) challenged (*Clostridium*/*Eimeria* co-infection) commercial broilers. Another in vivo study with the dietary supplementation of two garlic metabolites (10 ppm) namely propyl thiosulfinate (PTS) and propyl thiosulfinate oxide (PTSO) revealed increased body weight gain, decreased fecal oocyst excretion, a higher profilin antibody response, and greater spleen cell proliferation in *E. acervulina*-infected chickens as compared with the infected birds fed a non-supplemented control diet [73]. Ali et al. [91] also reported that the dietary supplementation of garlic powder (15g/kg of feed) reduced oocysts shedding and lesion score as well as lowering mortality, and improved histopathology of the small intestines in the supplemented group. They ascribed these effects to the presence of allicin and phenolic compounds in garlic. The allicin has antioxidant and antiparasitic activity which directly kill the sporozoites [73]. The phenolic compounds in garlic act on the cytoplasmic membrane of *Eimeria* and make changes in their cation permeability, leading to the death of pathogens [91].

Salem et al. [27] assessed the efficacy of garlic extract (40 mg/mL) in experimentally *S. typhimurium* induced salmonellosis in Cobb broiler chicks. The garlic extract used in the study contained allicin, alliin, allylsulfide, E-ajoene, and vinyldithiin. The mortality rate was decreased from 53.3% to 13.3% after treatment with garlic extract. The body weight of the infected chickens was significantly improved with the treatment of garlic extract when compared with infected non-treated groups. The post-mortem lesions were less severe in the garlic-treated infected chicks as compared with control infected chicks. The authors suggested the efficacy of garlic against multidrug resistant *Salmonella* by reducing its invasion, resistance to antimicrobial agents, and biofilm formation ability. Jimoh et al. [90] reported that garlic at the various supplementation levels reduced the caecal load of *C. perfringens* as compared with the control group and attributed to the OSCs.

Kavindra and Shalini [92] reported in vitro anthelminthic potential of garlic oil (2%, 4%, and 6%) against *Ascaridia galli* diseases in poultry birds. Mechanistically, the garlic oil reduced significantly the glucose uptake, glycogen content, oxygen consumption, and relative activity of acid and alkaline phosphomonoesterases in the parasite. However, an in vivo study by Velkers et al. [28] failed to observe efficacy of allicin from garlic against experimentally induced *A. galli* infection in chickens with no significant effect on worm load. Shojai et al. [93] observed an inhibitory effect of garlic extract against infectious bronchitis virus in specific pathogen-free (SPF) embryonic eggs and they suggested that the garlic extract could have an effect on the virus in replication phase. From the above discussion, it can be inferred that allium compounds at a certain inclusion rate can alleviate the negative effects of infections in chickens and mediate multiple disease-related signaling pathways.

### 3.4. Effects on Intestinal Microbiota and Morphology

The gastrointestinal tract of poultry harbors complex assemblages of microorganisms (microbiome) mainly dominated by the phyla Firmicutes (*Lactobacillus, Streptococcus*, *Bacillus*, *Enterococcus*), Bacteroidetes (*Bacteroides*, *Bifidobacterium*), Proteobacteria (*Escherichia*, *Salmonella, Campylobacter, Shigella)* and Actinobacteria [94]. This gut microbiome is recognized as a key player in governing host growth performance and health by providing nutrients from indigestible dietary substrates, competitive exclusion of pathogens, detoxification, strengthening the gut barrier, and modulation of immune system [95,96]. Pan and Yu [97] suggested an intertwined relationship of the gut microbiome with poultry host and diet. Therefore, any perturbation in the taxonomic composition of gut microbiota (called dysbiosis) may underlie its contribution to symptoms of a disease condition like that in humans. Recently, few studies strengthened the applicability of alliums (mainly garlic and onion) as poultry feed additive in the gut microbiota modulation in regard to diversity and composition [53,54,74,75,80,98]. Supplementation of onion showed a significant reduction in the population of *E. coli* and increased significantly *Lactobacillus* and *Streptococcus* species. Similarly, Goodarzi et al. [99] and Shargh et al. [100] also reported higher *Lactobacilli* spp. and reduced *E. coli* load in ileum of onion fed broilers. Shin et al. [101] hypothesized that the phylum Proteobacteria may potentially serve as biomarker for gut dysbiosis in humans. Intriguingly, Kim et al. [102] demonstrated that lower numbers of certain gut pathogens such as *E. coli* may improve broiler performance. Sheoran et al. [56] and Kirubakaran et al. [57] also indicated that the lower *Staphylococcus aureus* and *E. coli* as well as aflatoxins producing fungi in the intestine fostered nutrient digestibility which in turn improve weight gain of the birds. 

Allicin has also been reported to improve and regenerate the physiological structure of the intestinal epithelium layer, and enhance crypt depth and villus height, which ultimately support its digestive capacity through increased absorption of nutrients and assimilation [103]. However, the instability and poor bioavailability of allicin question its effects in vivo [23]. Ur Rahman et al. [53] observed that onion supplementation significantly increased dimensions (villus height, width, crypt depth, and surface area) of duodenum, jejunum, and ileum. The authors hypothesized that larger intestinal villi are associated with higher absorption of the nutrients and reduction of *E. coli* in the intestine. Mehmood et al. [104] also reported that supplementation of onion in the feed significantly increased villus height, crypt depth, and surface area of the jejunum in broilers.

Karangiya et al. [80] indicated that garlic supplementation (10g/kg feed) increased the absorptive surface area of the intestine (villus height, width, and crypt depth) and correlated with the higher body weight gain in broilers. Diets containing garlic-derived propyl propane thiosulfonate (PTS-O) (0.045 and 0.090 g/kg feed) has also been shown to improve absorption surface at the ileal level in broilers [74]. In an extended study, Peinado et al. [75] observed a decrease in the numbers of enterobacteria, in particular lactobacilli and an increase in bacteroides in the broiler intestine with the dietary supplementation of PTS-O (0.045 and 0.090 g/kg feed). Although generally regarded as a beneficial group, the higher number of lactobacilli is linked to the impairment in fat digestion or absorption in poultry due to their bile-deconjugation activity [105,106]. Thomas et al. [107] suggested that the higher bacteroidetes was responsible for the improved performance in chickens since bacteroidetes are involved in fermentation of high molecular weight carbohydrates, activation of T-cell mediated immune responses, prevention of potential pathogens, bile acid metabolism, and transformation of toxic and/or mutagenic compounds. Likewise, Ruiz et al. [98] observed lower diversity indices of ileal mucosa-associated microbiota in chickens fed the PTS-O–supplemented diet, which was ascribed to the bactericidal effect of PTS-O against enterobacteria, coliforms, *E. coli*, *C. jejuni*, and *Salmonella* spp., as also observed by Peinado et al. [75]. In addition, PTS-O was able to significantly increase and modulate the composition of bifidobacteria in growing broilers; which are considered as excellent candidates of probiotics in broilers [108]. Another study involving PTS-O supplementation indicated negative correlations between relative abundances of *Escherichia*–*Shigella* or enterobacteria (crop, ileum and caeca) and growth performance as well as fat digestibility in PTS-O fed broilers [109]. When garlic extract (0.04 or 0.06 g/kg feed) was gavaged to broilers reduced number of *E. coli* and *Staphylococcus aureus* in the ileo-caecal digesta and improved nutrient digestibility were observed [110]. Kırkpınar et al. [111] reported that garlic oil alone or in combination with oregano, reduced *Clostridium* counts in the ileum of broilers. However, total organism, *Streptococcus*, *Lactobacillus* spp., and coliform counts were not affected by the dietary treatments. The lower *Clostridium* counts were ascribed to the antibacterial effects of essential oils. 

Notwithstanding the fact that specific mechanistic studies how dietary alliums affect chicken gut health and physiology are limited; it is clear from the above-cited findings that alliums participate in gut homeostasis to foster an intestinal environment conducive to commensals by reducing the expansion of pathogenic microorganisms. However, a better understanding of the gut/microbe interactions and gut microbial diversity using next generation sequencing will provide new opportunities for the improvement of poultry health and production.

### 3.5. Effects on Immune Response

Poultry diet and nutrition are critical determinants of birds’ immune response. Several studies have advocated disease prevention or immune enhancing effects of alliums in poultry, however, very few studies investigated the underpinning mechanisms for their specific immunomodulatory effects. For instance, Kim et al. [73] investigated the effects of two garlic secondary metabolites (10 ppm) namely PTS and PTSO on the in vitro and in vivo parameters of chicken gut immunity during experimental *E. acervulina* infection. In vitro, PTSO/PTS treatment dose-dependently killed invasive *E. acervulina* sporozoites and stimulated splenocyte proliferation. In vivo feeding of PTSO/PTS provided increased protective immunity following live *E. acervulina* challenge infection, as indicated by improved bodyweight gains, reduced fecal oocyst shedding, and higher anti-profilin serum antibody titers, compared with the non-supplemented controls. In PTSO/PTS-fed birds, microarray hybridization identified 1227 transcripts, whose levels were significantly altered (552 up-regulated and 675 down-regulated) in the intestinal intraepithelial lymphocytes (IEL) involving immune- and cardiovascular-related gene pathways and networks. The authors observed a simultaneous and interactive effects of PTSO/PTS dietary supplementation on adaptive (increased splenocyte proliferation and anti-prolifin titers) and innate immunity [downregulation of toll-like receptors (TLR) and nuclear factor kappa-light-chain-enhancer of activated B cells (NF-κB)] in chickens against coccidiosis. 

Hanieh et al. [112] reported that the dietary alliums (garlic and onion) have a potential to enhance the humoral immune functions in White Leghorn chickens following immunization with Newcastle disease virus (NDV), sheep red blood cells (SRBC) and *Brucella abortus* (BA). The authors observed that alliums (10 g/kg feed) enhanced anti-NDV, anti-SRBC, and anti-BA antibody production, which might be due to increased CD4/CD8 cells, following immunization. Moreover, the relative weight of spleen and thymus were increased in case of garlic supplementation, which was ascribed to the enhanced lymphocyte proliferation and the increase in WBC counts. The mechanism of improved humoral immune functions by the dietary alliums against three antigens was further delineated by a subsequent in vitro study on the lymphocytes and peritoneal macrophages from white Leghorn chickens (male) [113]. The authors observed that garlic and onion extract augmented concanavalin A (ConA)-induced splenocyte and thymocyte proliferations, and gene expression of IL-2 and IFN-γ as well as higher microbicidal activity and reactive oxygen species production in macrophages. They speculated different mechanisms of immune modulation by garlic and onion. Garlic had a direct stimulatory effect on the immune cells, whereas onion had an indirect stimulatory effect, the antioxidant activity of high flavonoids of onion may be a plausible explanation [112,113]. In contrast, Jafari et al. [114] and Goodarzi et al. [54] failed to report any significant effect on antibody titers against NDV in garlic and onion fed broilers, respectively.

Another study investigated the effect of dietary supplementation of *A. hookeri* on the inflammatory immune activities in the jejunum during the immunological stress induced by *Clostridium*/*Eimeria* co-infected commercial broilers [71]. The authors observed downregulated expression of pro-inflammatory cytokines such as IL-1β, IL-8, IL-17A, inducible nitric oxide synthase (iNOS), and lipopolysaccharide induced TNF-α (LITAF) in jejunum of NE challenged group by the dietary supplementation of *A. hookeri* as compared with control. In addition, the dietary supplementation of *A. hookeri* significantly increased expressions of tight junction (TJ) proteins [junctional adhesion molecule (JAM), occludin, and zonula occludens 1 (ZO1)] and intestinal mucin 2 (MUC2). These proteins play crucial roles in the regulation of intestinal permeability and barrier function [49].

Garlic dietary supplementation modulated chicks’ innate immune response via various mechanisms including phagocytosis augmentation, bactericidal activity enhancement and nitric oxide (NO) production reduction, together with triggering the IL-1β, IL-6 and IFN-γ cytokines expression levels in comparison with the non-supplemented chicks against multi-drug resistant *E. coli* O78 challenge [89].

Lee et al. [49] reported that the dietary supplementation of *A. hookeri* promoted gut integrity and enhanced innate immunity during an immunological stress induced by lipopolysaccharide (LPS) in young broiler chicken. They observed decreased levels of alpha-1-acid glycoprotein (α-1-AGP), a marker for systemic non-specific inflammation or gut barrier health. In LPS-challenged groups, chickens fed diets supplemented with *A. hookeri* (1 or 3g/kg feed) exhibited lower transcript levels of pro-inflammatory cytokines (IL-1b, IL-8, TNF superfamily member 15, and LITAF) as compared with the non-supplemented fed chickens. Furthermore, the dietary supplementation of *A. hookeri* significantly upregulated the expression levels of TJ proteins (JAM, occludin, and ZO1) and MUC2. 

Garlic powder and holy basil leaf powder either alone or in combination in the broiler’s diet have potent immune modulating activity by showing a stimulatory effect on relative mRNA expression of TLR 2, TLR 4, and TLR 7 in the commercial broilers [56]. However, Toghyani et al. [14] reported no influence of dietary garlic (4g/kg feed) on immune-related parameters such as antibody titers, lymphoid organs’ weight, albumin to globulin ratio, and heterophil to lymphocyte ratio in broilers and speculated that a higher dose is required to elicit any immune response. Indeed, the aforementioned data related to the immune effects of dietary alliums in poultry form the basis of further studies involving the mechanisms of molecular signaling and immune response initiation. Therefore, in a more long-term perspective, the assessment of variation among immune system components of poultry in response to allium supplementation will offer a better understanding of nutritional immunomodulation to reduce risk and manage field infections.

### 3.6. Effects on Product Quality

Dietary strategies are valuable options to improve nutritional value as well as oxidative stability and sensory properties of poultry meats and eggs. The antibacterial, anticoccidial, antifungal, antiviral, and antioxidant activity, as well as the immune-enhancing activity of allium-derived compounds have garnered attention in improving the poultry product quality. The plasticity and extraordinary responsiveness of poultry eggs to dietary factors make them the most attractive targets for nutrition modulation [115]. Several studies reported the use of alliums toward the improvement of egg quality [67,69,81,82,83,85]. Ao et al. [69] found a better fatty acid profile in egg yolk with higher poly-unsaturated fatty acid and lower saturated fatty acid by the dietary garlic (30 g/kg feed). Damaziak et al. [116] indicated that administration of the dietary onion extract to hens resulted in heavier eggs, with a higher content of egg yolk and better quality of albumen. The genus *Allium* has an exceptional ability to absorb, metabolize, and store selenium as organoselenium compounds such as selenomethionine and selenocysteine [117]. Olobatoke and Mulugeta [67] had given the possible explanation to the increased egg weight in laying hens is the absorption of garlic compounds (selenomethionine and selenocysteine) and their subsequent deposition in the egg yolk. Additionally, alliums are rich source of polyphenols (gallic acid, ferulic acid, quecetin, kaempferol, and flavonoid glycosides), potent antioxidants. The Haugh unit, albumen height, and pH are the indicators of the freshness of eggs, which tends to decrease during storage. Lim et al. [76] reported that with the dietary garlic in layers, the Haugh unit was improved during storage possibly due to the antioxidant effect from allicin and organoseleniums. Allicin inhibits the formation of superoxide by the xanthine/xanthine oxidase system, probably via a thiol exchange mechanism [118]. Wakebe [119] reported that the inclusion of selenomethionine in the layer diet (0.3 ppm/kg feed) resulted in higher Haugh units, which was ascribed to increased glutathione peroxidase activity in the egg yolk and white. Mahmoud et al. [85] proposed another explanation in that the garlic enhances the egg’s antioxidant status by upgrading the glutathione peroxidase activity in yolk and albumen; this thereby increased egg quality during storage with better albumen height, Haugh unit, and pH probably because of less lipid and protein oxidation. In an organoleptic assessment, Motozono et al. [120] reported an off flavor in eggs with garlic dietary supplementation (20 g crushed garlic/kg feed) in layers, while Birrenkott et al. [121] and Olobatoke and Mulugeta [67] reported no differences in color and flavor in eggs from hens consuming up to 30 g dietary garlic powder per kg feed. Damaziak et al. [116] indicated that the effect of dietary allium on the taste of eggs may be determined by both supplementation level and duration.

The inclusion of alliums in poultry diets has also been reported to improve color stability, fatty acid composition [63], sensory properties [14,111], and the anti-oxidative ability of meat [64,66,79]. Choi et al. [63] reported the color stability of meat (highest redness and yellowness) by the incremental levels of garlic powder (1–5%) dietary supplementation and the effect was ascribed to the reduced metmyoglobin formation and oxidation in thigh muscle of chicks. The same study observed better fatty acid profile in garlic supplemented groups by protecting the oxidation of unsaturated fatty acid. However, Abdullah et al. [122] reported no effects of garlic supplementation on the meat quality such as cooking loss percentage, shear force, color coordinates). The improved anti-oxidative capability of chicken meat by the dietary allium supplementation was attributed to the accumulation of antioxidant compounds such as flavonoids and OSCs [66,79]. Indeed, the utilization of dietary alliums to improve the quality of poultry products should be done carefully because high doses of the allium may reduce overall acceptability with altered taste and odor.

## 4. Factors Determining the Effectiveness of Alliums in Poultry Feed

Several variables need to be considered while recognizing the efficacy and safety of alliums in poultry (Table 2). From the above-cited studies, it is evident that six different kinds of allium formulations were mainly used in poultry, i.e., powder (sun- or air-dried), juice, purified extract, oil, aged extract, and paste. These processed alliums contain a variety of OSCs (major bioactive constituents) which differ greatly from their intact forms, depending on their manufacturing process. Most of these preparations were not chemically characterized and thus cannot be generalized under a single umbrella to have a biological response in poultry. Thiosulfinates, the most bioactive OSCs, are volatile and can evaporate rapidly, leading to largely varied final concentrations in the feed [123]. The pungent smell of thiosulfinates [21] might also affect the feed palatability, depending on the applied dosage. In the published literature, the inclusion rates of alliums in poultry have been reported to be very wide ranging from 0.001% to 10% (Table 1). For instance, Aji et al. [62] reported ineffectiveness of a low dose (25 and 50 mg of allium/kg feed) of onion and garlic supplementation while Varmaghany et al. [58] indicated negative effects of a high dose of garlic supplementation. Therefore, identification of an optimal dosage of alliums will also determine its effectiveness in poultry. Fujisawa et al. [124] reported that thiosulfinates might lose antimicrobial activity by reacting with sulfhydryl (SH) compounds of other feed components (proteins). The thermally unstable nature of allium bioactive constituents [16,20] also affects their application in feed production, since thermal processing is an important step to decontaminate the harmful microorganisms of feed. The OSCs have poor water solubility [125], which further limits their application in feed. While considering these factors, the higher cost of allium-based feed cannot be overlooked. Apart from these, the poultry responses might also be affected by various factors such as the feed type (pellet or mash) and quality, duration of study, hygiene, subject recruitment (broiler, layers, quails, etc.), age, health status (healthy or challenged) and environmental factors among many others [126]. Indeed, without proper standardized formulation, in practice the choice of an economically feasible allium-based feed additive is compromised in poultry diets.

## 5. Fortification/Preservation of *Allium* Bioactivity

The instability and volatility of allium bioactive compounds prompted animal nutritionists to devote intensive efforts in the search for new stabilization techniques that could ensure feed safety and quality as well as enhance modern preservation methods in the feed industry.

### 5.1. Fermentation

Fermentation has significantly improved bioactivities and organoleptic properties of alliums (Table 3). Allium fermentation resulted in higher polyphenolic content via the deglycosylation of complex phenolic glycosides to their simpler derivatives by the action of glucosidases, thereby increasing their antioxidant activity as well as bioavailability [127,128,129]. Fermentation could also reduce the pungent smell of alliums and hence expected to improve the palatability of feed [128,129,130]. Furthermore, fermented alliums can act as a viable source of probiotics and provide host health benefits [131]. Bernaert et al. [132] hypothesized that fermentation can be used as a stabilization technique for the preservation of antioxidant activity in *A. ampeloprasum* var. *porrum*. Hossain et al. [133] reported an increase in feed intake with the fermented garlic supplementation as compared with the control diet in broilers. Thus, the allium ‘probiotication’ may offer a cost-effective approach in the manufacture and storage processes of a feed additive by extending shelf-life and maintaining desired sensory properties in addition to the host health benefits. 

### 5.2. Microencapsulation/Nanotechnology

Microencapsulation is one of the most effective approaches for protecting bioactive compounds against oxidation, heat and evaporation, controlled delivery, uniform distribution, storage stability, masking off-flavours, and extending the shelf life without affecting their physical, chemical or functional properties [143]. Milea et al. [143] and Akdeniz et al. [144] successfully encapsulated phenolic compounds extracted from onion skin. Piletti et al. [145] reported that β-cyclodextrin encapsulation of garlic oil increased thermal stability and water solubility, as well as preserved antimicrobial activity. However, the use of microencapsulation is based on several factors, including feasibility, practicability, and cost [146].

Nanoparticles can be used as possible feed supplements for poultry to improve overall health and feed conversion ratio [147]. The formulation of plant-derived bioactive compounds using nanotechnology may result in their improved activity at low dosage [148]. Sundari et al. [149] reported turmeric extract nanoparticle as a feed additive which improved meat quality at low dosage without affecting performance in broilers. Xu et al. [125] converted natural organosulfur compounds into nanometer-sized iron sulfides (nFeS) with improved antibacterial activity and antibiofilm efficacy in vitro. Jini and Sharmila [150] synthesized silver nanoparticles from *A. cepa*, which have higher in vitro antidiabetic and antioxidant activities. However, the toxicity of nanoparticles due to nano size and the high cost hinder their practical application in poultry.

## 6. Future Perspectives

The aforementioned findings are testimony to the fact that the appreciation of alliums as poultry feed additives exhibits tremendous opportunities as well as hurdles. Therefore, the scientists, veterinarians, and commercial partners must work together to thwart the limitations for optimal efficacy of alliums, from poultry health and economic perspectives. Future research in this field will help us to better understand their mechanism of action and optimal dosage as well as efficient delivery methods (fermentation, microencapsulation and/or nanotechnology). Since alliums are a hub of bioactive compounds which might affect poultry production synergistically, the dietary supplementation of dried alliums or their extracts poses an advantage over the single extracted compound. Moreover, the synergistic effect of alliums with other antibiotic alternatives such as prebiotic, probiotic, organic acid, etc. together with good management and farming practices will be the key to achieve sustainable poultry production. Moreover, researchers in this field should be encouraged to publish even the negative or no effects of alliums in poultry.

## 7. Conclusions

It is evident from our discussion that alliums harbor a variety of bioactive compounds such as organosulfur compounds, flavonoids, fructans, fructo-oligosaccharides, saponins, etc., and thereby justifying their usefulness as feed additives for poultry production. Recently, several studies have established that alliums in poultry diets have a significant modulatory effect on their growth performance indices, lipid metabolism, gut ecosystem as well as immune responses, especially when poultry are experiencing stress and disease challenge conditions. In addition, the alliums also improve the nutritional quality of poultry products via their enrichment in antioxidant (flavonoids) and organoselenium compounds. However, their application in poultry production has been largely circumvented due to inconsistent efficacy among studies, lack of a clear understanding of the mechanisms of action, non-availability of a standard as well as chemically characterized formulation, and higher cost. The processing methods, such as extraction, encapsulation, fermentation, and heating strongly influence the chemical composition, ergo, the biological activity of alliums. Therefore, poultry nutritionists must understand the inherent differences among the allium products used in various studies, along with their potential role in providing desired potential effects when added to poultry feed. A standardized procedure should be developed for an allium-based feed additive retaining its bioactive components. The OSCs and polyphenol contents of allium products may serve as proxy for their strategic application in poultry nutrition feeding programs. In addition, to preserve the effectiveness of alliums as a poultry feed additive, the optimization of dosage regimens that encompasses bioavailability could also be a suitable strategy. This review is expected to inspire investigations on alliums as feed additives for poultry health and disease management.

## Figures and Tables

**Table 1:** Effect of dietary supplementation of *Allium* spp. in poultry.

Animals	*Allium* spp. & treatments	Formulation	Main active components	Effects	Ref.
Male Shaver Starbo broilers (7-d-old)	0.5 or 5 g *A. sativum*/kg feed; 8 weeks	Raw and boiled Powder	ND	↑ BWG; ↑ Oxidative stability of meat during storage	[64]
104 Mixed sex broilers (4-week-old)	*A. cepa* (0, 0.025, 0.05, or 0.1 g) and *A. sativum* (0, 0.025, 0.05, or 0.1 g)/kg feed; 3 weeks	Powder	ND	↑ BWG; ↑ Feed and water intake; ↑ FCR	[62]
320 Ross-308 broilers (1-d-old)	1.5, 2.0, or 2.5 g *A. cepa*/kg feed; 6 weeks	Powder	ND	↑ Body weight; ↑ Feed intake; ↑ *Lactobacillus* and *Streptococcus*; ↓ *E. coli*; ↑ Intestinal histomorphology	[53]
60 Ross broilers (1-d-old) (Challenged by live *Eimeria acervulina*)	0.01 g Garlicon40^®^ (*A. sativum*)/kg feed; 20 days	Powder	67% Propyl thiosulfinate and 33% propyl thiosulfinate oxide	↑ BWG; ↓ Fecal oocyst shedding; ↑ *E. acervulina* profilin antibody responses	[73]
144 Male Cobb broilers (1-d-old)	0.045 or 0.09 g Proallium-SO-DMC^®^ (*A. sativum*)/kg feed; 3 weeks	Powder	11.3% Propyl propane thiosulfonate	↑ Body weight; ↓ Feed intake; ↓ Feed/gain ratio; ↓ Enteropathogens; ↑ Ileal histological structure	[74]
144 Male Cobb broilers (1-d-old)	0.045 or 0.09 g Proallium-SO-DMC^®^ (*A. sativum*)/kg feed; 3 weeks	Powder	11.3% Propyl propane thiosulfonate	↑ Nutrients digestibility; Modulate intestinal microbiota	[75]
300 Male Arbor Acres broilers (1-d-old)	10–50 g *A. sativum*/kg feed; 5 weeks	Powder	ND	↓ Total serum cholesterol levels; ↓ LDL-C; ↑ HDL-C; ↑ Meat quality (↑ Color stability; ↓ TBARS; ↑ Total unsaturated fatty acid:total saturated fatty acid ratios)	[63]
150 Hy-Line Brown layers (50-week-old)	10–50 g *A. sativum*/kg feed; 5 weeks	Powder	ND	↑ Haugh Unit; ↓ Total serum cholesterol; ↓ Total egg yolk cholesterol	[76]
24 Mixed sex broilers (1-d-old)	15 or 30 g *A. sativum*/kg feed; 8 weeks	Powder	ND	Regulate lipid metabolism (↓ Plasma cholesterol; ↓ triglycerides; ↓ VLDL-C; ↓ LDL-C; ↑ HDL-C)	[77]
150 Male Ross-708 broilers (1-d-old) (Challenged by LPS	10 g or 50 g *A. hookeri*/kg feed; 8 days	Powdered fermented roots	ND	↑ Body weight; ↓ Inflammatory response (↑ Expression of intestinal tight junction proteins and mucin; ↓ Serum α-1-AGP; ↓ Pro-inflammatory cytokines)	[49]
125 Male Ross-708 broilers (1-d-old)	10 g *A. hookeri*/kg feed; 3 weeks	Powdered roots	ND	↑ Body weight; ↑ antioxidant activity (↑Gene expression of heme oxygenase, aflatoxin B1 aldehyde reductase, SOD 1, and CAT; ↑Serum levels of SOD, CAT, and MDA)	[70]
300 male Ross-708 broiler chicks (1-d-old) (Challenged by NE)	10 g or 30 g *A. hookeri*/kg feed; 20 days	Powdered roots	ND	↓ Loss of BWG; ↓ Lesion; ↓ Fecal oocyst shedding; ↑ Innate immunity (↓ Pro-inflammatory cytokines; ↑ Expression of tight gut junction proteins and mucin)	[71]
500 Arbor Acres broiler chicks (1-d-old)	1–4 g *A. sativum*/kg feed; 5 weeks	Fermented powder	ND	↑ WBC, lymphocyte and IgG; ↓ Total cholesterol; ↓ Triglyceride; ↓ LDL-C; ↓ Cortisol; ↑ Meat quality (↓ TBARS and pH)	[66]
600 White mini broiler chicks (1-d-old)	3 or 5 mL *A. cepa*/kg feed; 5 weeks	Fermented liquid	ND	↑ Final body weight; ↑BWG; ↓ Serum cholesterol; ↓ Triacylglycerol	[78]
400 Male Ross-308 broiler chicks (3-d-old)	5, 7.5, or 10 g *A. cepa*/kg feed; 4 weeks	Liquid extract	ND	↑ BWG; ↑ Feed intake; ↑ ATTR; ↑ Serum IgG; ↓ TBARS	[79]
240 Cobb-400 broiler chicks (1 day old)	10 g *A. sativum*/kg feed; 6 weeks	Powder	ND	↑ BWG; ↑ Final weight; ↑ Villi length and width; ↑ Cryptal depth	[80]
288 commercial broiler chicks (1-d-old)	5 or 10 g *A. sativum*+1 or 2 g *Piper nigram*/kg feed; 6 weeks	Powder	ND	↑ BWG; ↑ Final weight; ↑ FCR	[57]
36 layers of six different strains (Hisex Brown, Isa Brown, Lohmann, Starcross, Babcock, and Starcross-579 strains) (28-week-old)	20, 40, 60, 80, or 100 g *A. sativum*/kg feed; 6 weeks.	Paste (sun-dried)	ND	↑ Egg production; ↑ Egg yolk weight; ↓ Egg yolk cholesterol; ↓ Serum cholesterol	[81]
162 SHSY-type brown layers (21-week-old)	5 or 10 g *A. sativum*/kg feed; 22 weeks	Powder	ND	↑ Egg weight; ↓ Egg yolk cholesterol; ↓ Serum triglyceride and cholesterol	[82]
300 Quails (9-week-old)	5 or 10 g *A. sativum*/kg feed; 21 weeks	Powder	ND	↑ Egg weight; ↓ Egg yolk cholesterol; ↓ Serum cholesterol	[68]
72 Dekalb white layers (30-week-old)	30 or 50 mg *A. sativum*/kg feed; 7 weeks	Powder	ND	↑ Albumen height; ↑ Haugh units; ↑ Egg and albumen weight	[67]
120 laying Japanese quails (10-week-old)	10, 20, or 40 g *A. sativum*/kg feed; 12 weeks	Powder	ND	↓ Egg yolk cholesterol; ↓ Serum cholesterol	[83]
240 Isa brown laying hens (41-week-old)	10, 20, or 40 g *A. sativum*/kg feed; 5 weeks	Fermented powder	ND	↑ Yolk height and color; ↑ Haugh Unit; ↓ Serum total cholesterol; ↑ Yolk PUFA:SFA ratio	[69]
108 laying hens (30-week-old)	5 or 10 g *A. sativum* and 10 g *A. cepa*/kg feed; 4 weeks	Powder	ND	↑ FCR; ↑ Egg production; ↑ Egg weight; ↓ Total cholesterol; ↓ Creatinine	[84]
180 Isa Brown hens (18-week-old)	10, 20, 30, 40, or 50 g *A. sativum*/kg feed; 20 weeks	Paste (Raw)	ND	↑ HDL-C; ↓ Total cholesterol; ↓ Yolk cholesterol; ↓ LDL-C	[65]
640 Mixed sex Ross 308 broiler chickens (body weight 45 ± 7 g)	1, 1.5, or 2.25 mL/kg feed; 6 weeks	Liquid	ND	↓ Mortality; ↓ FCR; ↑ EPEF; ↑ Dressing percentage; ↑ Weight of breast muscles; ↑ Liver weight; ↑ Protein and crude ash content of breast meat; ↑ Total protein content of serum	[55]

ND: Not determined; BWG: Body weight gain; FCR: Feed-conversion ratio; HDL-C: High-density lipoprotein-cholesterol; LDL-C: Low-density lipoprotein-cholesterol; VLDL-C: Very low-density lipoprotein-cholesterol; LPS: Lipopolysaccharide; α-1-AGP: Alpha-1-acid glycoprotein; SOD: Superoxide dismutase; CAT: Catalase; MDA: Malondialdehyde; NE: Necrotic enteritis; WBC: White blood cells; IgG: Immunoglobulin G; TBARS: Thiobarbituric acid reactive substances; ATTR: Apparent total tract retention of nutrients; PUFA: Polyunsaturated fatty acid; SFA: Saturated fatty acid; EPEF: European production efficacy factor.

**Table 2:** Factors determining the effectiveness of alliums in poultry.

Items	Characteristics	Effects
Thiosulfinates	Volatile	Varied final concentration in feed
	Pungent smell	Feed palatability
	Reacts with SH groups of other feed constituents	Loss of antimicrobial activity
	Thermal instability	Difficult in feed processing
	Poor water solubility	
Formulation	Variation in chemical constituents	Variation in biological response
Dosage	Pungent smell	Feed palatability
Other factors	Subjects (broilers, layers, quails, etc.), age, health status, feed type and quality, environmental conditions, and duration of study	Inconsistent results

**Table 3:** Fermentation of *Allium* spp. with respect to compositional changes and bioactivities.

Plants	Microorganisms	Fermentation Conditions	Compositional Changes	Study	Biological Activities	Ref.
*A. sativum*	*Saccharomyces cerevisiae*; *Lactobacillus plantarum*; *Mimulus pilosus*	*S. cerevisiae* and *L. plantarum* at 25 and 37 °C, respectively, for 48 h and *M. pilosus* at 25 °C for 7 days	↑ S-allyl-l-cysteine and cycloalliin; ↓ γ-Glutamyl peptide	-	ND	[130]
*A. sativum*	*L. plantarum*	37 °C for 24 h	Alliin content ↓; ↑ Cycloalliin content; ↑ S-allyl cysteine content	In vivo (mice model)	Lipid metabolism and antioxidant	[134]
*A. sativum*	Spontaneous	40 days at 60–70 °C and 85–95% relative humidity	↑ Polyphenol content	In vitro	Antioxidant	[135]
*A. cepa*	*Aspergillus kawachii* crude enzyme extract	30 °C for 24 h	↑ Quercetin and quercetin-3-glucoside; ↓ quercetin-3,4′-diglucoside and -4′-glucoside	In vitro	Antioxidant and neuroprotection	[136]
*A. cepa*	Spontaneous	37 °C for 3.5 days	Flavonoid profile changed	In vitro	Antibacterial, antigenotoxic, and antiproliferative	[137]
*A. cepa*	*Aspergillus oryzae*, *Bacillus subtilis*, *L. plantarum*, and *S. cerevisiae*	30 °C for 3 days	↑ Isoquercitrin	In vitro		[138]
*A. cepa*	Spontaneous	96–108 h with or without 1% (*w*/*v*) salt	↑ Lactic acid and acetic acid; ↓ amino acids; ↑ esters, alcohols, and aldehydes	In vitro	Flavor	[139]
*A. cepa*	*L. plantarum*	19 °C for 48 h	↑ Quercetin diglucoside	In vitro	Antioxidant	[140]
*A. ampeloprasum* var. *porrum*	*L. plantarum*, *Leuconostoc mesenteroides*, and *Lactobacillus sakei*	18 °C for 3 weeks	ND	In vitro	Sensory properties	[141]
*A. ampeloprasum* var. *porrum*	Spontaneous	18 °C for 21 days	↑ Polyphenol; ↓ methiin and isoalliin	In vitro	Antioxidant	[142]
*A. tuberosum*	*L. mesenteroides*	30 °C for 3 days	↑ Polyphenol	In vitro	Antibacterial and antioxidant	[127]
*A. chinense*	*L. plantarum*	37 °C for 7 days	↑ Polyphenol content; ↑ Free amino acid content; ↓ Sulfur containing compounds	In vitro	Antioxidant and flavor	[128]
